# A Study on a Novel Phase Change Material Panel Based on Tetradecanol/Lauric Acid/Expanded Perlite/Aluminium Powder for Building Heat Storage

**DOI:** 10.3390/ma9110896

**Published:** 2016-11-05

**Authors:** Enyu Wang, Xiangfei Kong, Xian Rong, Chengqiang Yao, Hua Yang, Chengying Qi

**Affiliations:** 1 School of Energy and Environmental Engineering, Hebei University of Technology, Tianjin 300401, China; wey@hebut.edu.cn (E.W.); shen8kobe24@gmail.com (C.Y.); yanghua@hebut.edu.cn (H.Y.); qicy@hebut.edu.cn (C.Q.); 2 School of Civil Engineering, Hebei University of Technology, Tianjin 300401, China; xrong@hebut.edu.cn

**Keywords:** phase change material panel, heat storage, tetradecanol, lauric acid, expanded perlite, aluminium powder

## Abstract

Phase change material (PCM) used in buildings can reduce the building energy consumption and indoor temperature fluctuation. A composite PCM has been fabricated by the binary eutectic mixture of tetradecanol (TD) and lauric acid (LA) absorbed into the expanded perlite (EP) using vacuum impregnation method, and its thermal conductivity was promoted by aluminium powder (AP) additive. Besides, the styrene-acrylic emulsion has been mixed with the composite PCM particles to form the protective film, so as to solve the problem of leakage. Thus, a novel PCM panel (PCMP) has been prepared using compression moulding forming method. The thermal property, microstructure characteristic, mechanical property, thermal conductivity, thermal reliability and leakage of the composite PCM have been investigated and analysed. Meanwhile, the thermal performance of the prepared PCMP was tested through PCMPs installed on the inside wall of a cell under outdoor climatic conditions. The composite PCM has a melting temperature of 24.9 °C, a freezing temperature of 25.2 °C, a melting latent heat of 78.2 J/g and a freezing latent heat of 81.3 J/g. The thermal conductivity test exposed that the thermal conductivity has been enhanced with the addition of AP and the latent heat has been decreased, but it still remains in a high level. The leakage test result has proven that liquid PCM leaking has been avoided by the surface film method. The thermal performance experiment has shown the significant function of PCMP about adjusting the indoor temperature and reducing the heats transferring between the wall inside and outside. In view of the thermal performance, mechanical property and thermal reliability results, it can be concluded that the prepared PCMP has a promising building application potential.

## 1. Introduction

With the people’s demands for indoor thermal comfort improvements, the energy used for space cooling and heating has been increasing in recent years, which can be exemplified by the fact that the operation and life-cycle building energy consumptions have accounted for about 20%–25% [1] and 43% [2], respectively, of total energy consumption in recent years. The heat storing/releasing characteristic of PCM has been used to enhance the thermal inertia of buildings and adjust the indoor temperature environment [3], and thus the building energy consumption can be reduced.

For the advantage of passively reducing the indoor temperature fluctuations, PCM has been widely studied to be incorporated within the building envelope, such as PCM wall [4,5,6], PCM wallboard [7,8,9] and PCM roof [10,11,12]. Although the combining patterns of PCMs with building envelopes are various, a key point in practical application is always the selection of appropriate PCM with high thermal property. The frequently-used PCMs in buildings are organic PCMs for their low supercooling, such as paraffin, fatty acid, ester, alcohols, glycols, etc. [13]. Among these researched PCMs, fatty acids, including capric acid, lauric acid, and so on, have attracted much attention in building applications. Besides, the phase change points of fatty acids can be lowered through two or more kinds of fatty acids mixing to form the eutectic mixture [14]. Lv et al. have studied the thermophysical property of eutectic mixture of capric acid and lauric acid (82 wt %:18 wt %), and tested their thermal performance [15,16,17]. Sarı and Karaipekli have prepared a series of form-stable composite PCMs based on eutectic mixtures of fatty acids [18,19]. Furthermore, a novel ternary eutectic mixture including capric–myristic–palmitic acids with a mass ratio of 64.8 wt %:22.6 wt %:12.6 wt % was developed by Yuan et al. [20]. Thereby, the fatty acids have been widely used to study the building heat storage.

Another important point for PCM building application is the PCM encapsulation. In early researches, PCMs were directly mixed with the building wall materials (concrete, mortar, etc.), but the serious leakage of liquid PCMs from wall materials can reduce the mechanical property of concretes or mortars [21,22]. To deal with this problem, before the combination with building envelopes, PCMs should be encapsulated. Due to the low cost and high flexibility, the method whereby PCMs are encapsulated into the micropores of supporting materials by vacuum impregnation method has received extensive attention. Sarı and his research team conducted a series of studies about the PCMs absorbed into porous building materials, and their researches have proved the feasibility and reliability of vacuum impregnation method [18,19,23,24]. Jiao et al. have developed a kind of composite PCM, in which the binary eutectic of lauric acid–stearic acid was impregnated into the expanded perlite under the vacuum condition [25]. Furthermore, Zhang et al. have fabricated the thermal-regulated gypsum board based on the composite PCM of capric acid–palmitic acid/expanded perlite, which was also prepared using vacuum impregnation method [26]. In the above researches, it was found that the composite PCM with vacuum impregnation have significant potential in building heat storage application. However, two difficult points to restrict the next research progress have emerged. Firstly, a slight leakage was found in the fabrication or application of composite PCM [27,28,29], and, without additional measures, the mass fraction of PCMs in the total composite materials should be reduced, in order to decrease the leakage [24]. Secondly, some supporting materials have the property of low thermal conductivity, which can be exemplified that the expanded perlite was the a kind of building insulation materials with a poor thermal conduction, and the low thermal conductivity can be considered is as essential disadvantage for PCM completing the melting–freezing cycle [24,30,31].

Aiming to improve the related research, a eutectic mixture with high latent heat and appropriate phase change point, which was composed of lauric acid and tetradecanol, has been investigated in this study, and it was encapsulated with expanded perlite to prepare a novel composite PCM using vacuum impregnation method. Furthermore, to deal with the reported problems, aluminite powder was added into the novel composite PCM to improve the thermal conductivity, because of reasonable cost and high thermal conductivity of aluminite powder, and, thus, additional measure of surface filming was applied in the preparation process, so as to avoid the leakage and obtain high PCM content. From the aspect of convenience in applying, a new kind of PCM panel was developed, based on the prepared composite PCM, through the method of compression moulding forming. As far as the authors are aware, there is no investigation reported in the literature on PCM panel with the raw materials of lauric acid-tetradecanol/expanded perlite-aluminite powder, and the surface filming and compression moulding forming processes. Besides, after the thermal performance test in cells under the outdoor climatic condition, the result indicated that this novel PCM panel has a significant opportunity to be used in buildings.

## 2. Experimental

### 2.1. Materials

**Figure 1:**
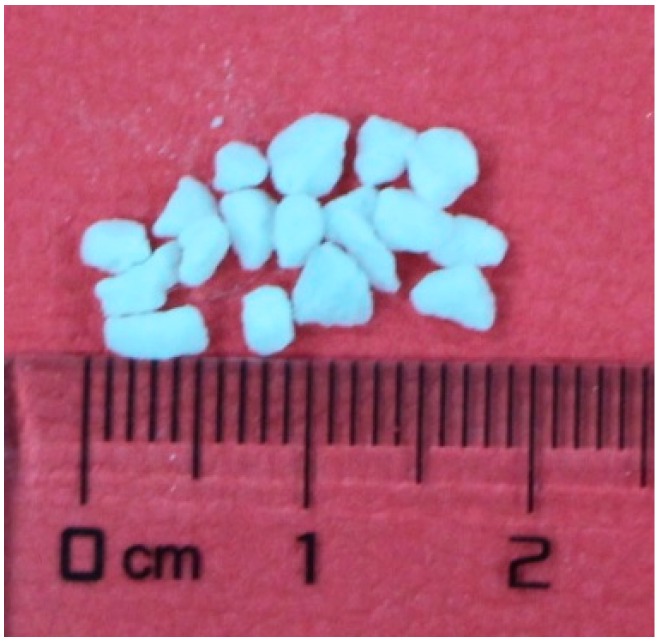
Appearance picture of EP.

The eutectic mixture of tetradecanol and lauric acid was chosen to be PCM in the preparation of the composite PCM, based on the consideration of safe, low cost, high thermal performance and suitable phase change point. As one kind of fatty acids, LA have pungent odour, but TD is odourless [32]. The mixing of LA with TD can release less pungent odour than mixing with other fatty acids or single LA in equalmass condition. TD and LA with the purity higher than 99.0% were purchased from Tianjin Yong Da Chemical Reagents Research Center (Tianjin, China). As shown in Figure 1, EP (particle size range: 2~5 mm; density: 192 kg/m^3^; water absorption: 380%; cylinder compressive strength: 86%; porosity: 62%) was obtained from Shang Tian Ti Mining Company (Xinyang, China). The chemical composition of EP has been presented in Table 1. AP with particle size of 18 μm was supplied from Tianjin Le Tai Chemical Co., Ltd. (Tianjin, China). Before using, both EP and AP were dried under the temperature of 105 °C for 24 h.

### 2.2. Preparation of Novel Composite PCM

#### 2.2.1. Binary Eutectic Mixture

The phase change point is an important factor for building application of PCM. Binary eutectic mixture maintains the same stability with its components, and has a lower melting temperature than that of the two components [33]. Thus, a kind of PCM with appropriate phase change point can be prepared through binary eutectic mixing. Two methods, proportioning test and theoretical prediction were usually used to obtain the optimum proportion of binary components in preparation binaryeutectic mixture [16,34]. Proportioning test refers to that the binary mixtures (A–B) with a series of component proportions (wt), such as A 95%:B 5%, A 90%:B 10% and so on, were measured by DSC, and then an optimum proportion will be ascertained by comparing the DSC curves. The proportioning test method can obtain the result with relative accuracy, but it needs more number tests and costs more time than the theoretical prediction method. Based on the accurate phase change data for the binary components, the precise proportion can also be achieved through theoretical prediction method [14]. In this study, the component proportion of TD-LA binaryeutectic mixture was ascertained by the theoretical prediction.

According to the second law of thermodynamics and phase equilibrium theory, the liquid phase equilibrium equation [14] of TD-LA binaryeutectic system can be obtained as Equation (1).

(1) $$-\frac{H_{i}}{T_{i}}(T_{m}-T_{i})+RT_{m}\ln(1-X_{i})+G_{i}=0$$

where subscript *m* and *i* denote the eutectic mixture and one of components (TD or LA) in the mixture; *H* and *T* are the latent heat of fusion and melting temperature of PCM, respectively; *R* is the gas constant (8.315 J·k^−1^·mol^−1^ in this study); *X* indicates the molar fraction of each component in binaryliquid mixture, and *X_TD_* + *X_LA_* = 1; and *G* is the excess free enthalpy of PCM.

For the commonly researched PCMs, especially alkanes, fatty acids and high alcohols in organic PCMs, the excess free enthalpy (*G*) can be assumed to be zero in Equation (1). Then, the melting temperature of eutectic mixture (*T_m_*) and the mass percentages for TD and LA in mixture were obtained from Equation (2). The calculated values and the comparison with measured values were listed in Table 2, in which, a good agreement was shown between the predicted melting temperature and the measured value, and the error was 6.2%. The small difference between measured and calculated values has validated the accuracy of theoretical prediction used in preparation of TD-LA binaryeutectic mixture.

(2) $$T_{m}=[\frac{1}{T_{i}}-\frac{\left(R\ln X_{i}\right)}{H_{i}}{]}^{-1}$$

#### 2.2.2. Novel PCMP

**Figure 2:**
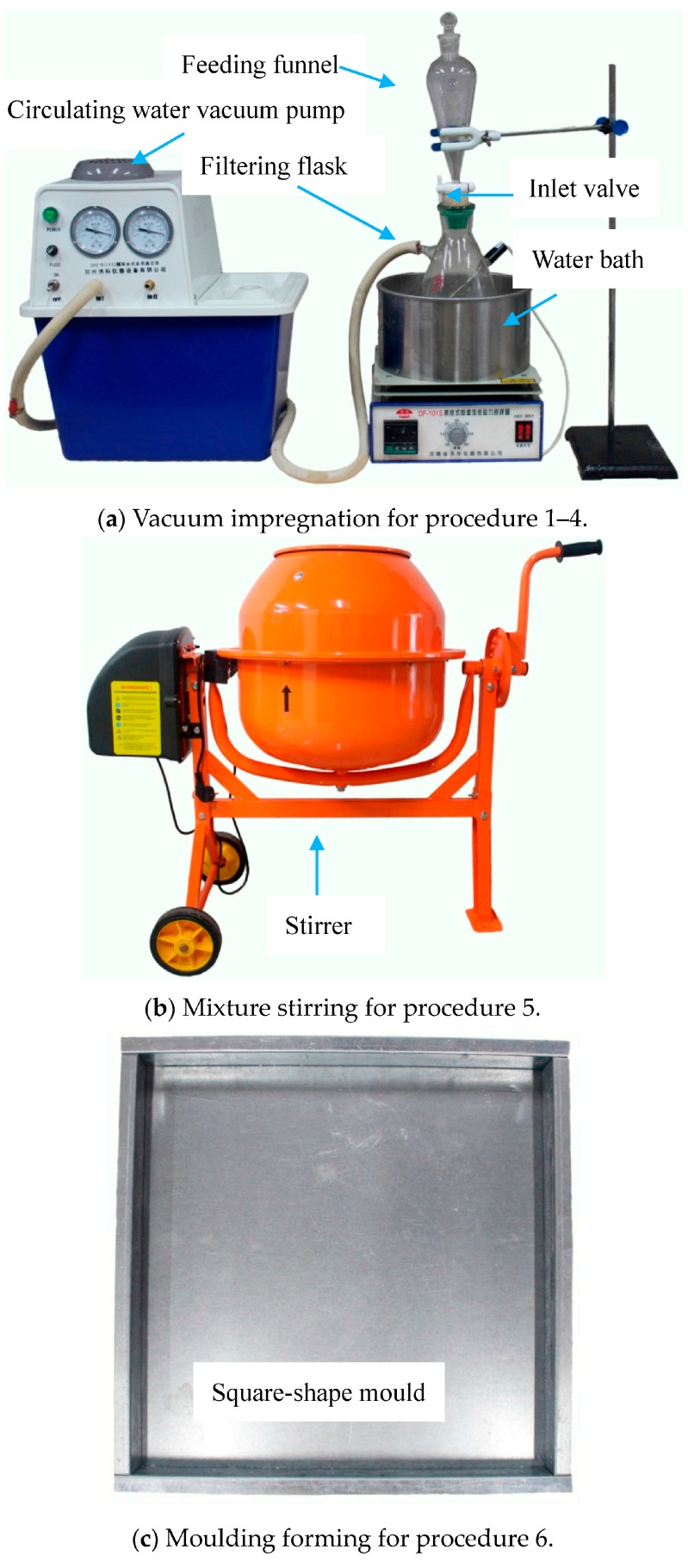
Schematic diagram of the novel PCMP preparation.

The novel PCMP was prepared by vacuum impregnation method [18,19], mixture stirring and compression moulding forming. The detailed procedures of preparation have been demonstrated in Figure 2 and summarized as the following.

**Figure 3:**
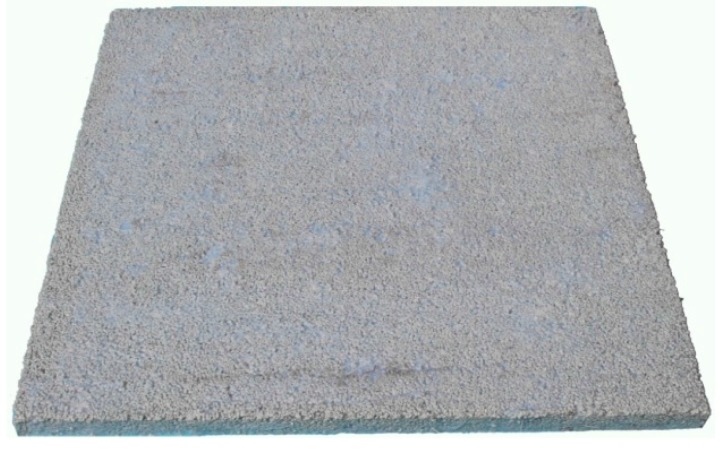
External view of prepared PCMP.

(1) TD and LA with a mass proportion of 53.60%:46.40% were mixed, melted and stirred in a vessel that was heated by the water bath for 30 min under 60 °C, and then the melting TD-LA mixture was poured into a feeding funnel in the top of a filtering flask, the inlet valve of which remained closed.
(2) EP was placed into the filtering flask, in which the internal space was maintained to be vacuum state and the inside air of EP’s porous structure was evacuated by a circulating water vacuum pump.
(3) After the inlet valve opened, the liquid of TD-LA flowed into the feeding funnel and mixed with EP through magnetic stirring. Because of the strong capillary action under vacuum state, the liquid TD-LA can be absorbed into the micropores of EP. The mass fraction ratio of TD-LA vs. EP was 50%:50%.
(4) The vacuum process was continued for 2 h at vacuum pressure of 65 kPa, and the mixture of TD-LA and EP in the filtering flask was heated by water bath, so as to maintain TD-LA melting in the vacuum process. Due to achieving the full penetration of TD-LA into EP, the air was allowed into the filtering flask to force the liquid PCM to further penetrate into the micropore structures of EP, when finishing the vacuum process.
(5) The low thermal conductivity of TD, LA and EP possibly leads to melting-solidifying circulation incompletion [35]. In order to enhance the thermal conduction of TD-LA/EP, AP was added and uniformly mixed with TD-LA/EP through stirrer (Figure 2b). Then, the styrene-acrylic emulsion was also put into the stirrer to form the film attached on the TD-LA/EP-AP surface.
(6) The styrene-acrylic emulsion was also the binding element for the PCMP. When finishing the stirring process, the composite TD-LA/EP-AP with styrene-acrylic emulsion was compacted to the novel PCMP through the square-shape mould, as shown in Figure 2c. When the novel PCMP was completely dry, the mould was removed. Finally, the novel PCMP has been prepared well, as shown in Figure 3.

### 2.3. Property Test Methods

The thermal properties of novel composite PCM, inclusive of phase change points and latent heats, were measured by DSC (STA409PC model by NETZSCH, Ahlden, Germany), with a constant heating rate of 5 °C/min [18,19] in the interval 10–100°C and the protection of purified nitrogen atmosphere at atmospheric pressure.

The microstructure and surface morphology for the novel composite PCM was researched by scanning electronic microscope (SEM) (S-3400N model by Hitachi, Tokyo, Japan) with two kinds of observation scales of 30 μm.

The thermal conductivities of novel composite PCMs were measured by the guarded plate method (DRH-III model, measurement range of 0.01–3.00 W/(m^2^/K), accuracy of ±2%), in which the thermal conductivities was obtained through measuring and calculating the data of heat flux and temperature differences between surfaces, on account of the one-way steady heat conduction principle. The heat transfer coefficient (U) of PCMP was obtained through heat flow meter method [36] under the same condition of one-way steady heat conduction.

Thermal cycle tests with the cycles of 1000 and 2000 times were conducted using two thermostatic water baths as the cold and heat source, respectively. A thermal cycle consisted of melting process with the heat source of 50 °C and freezing process with the cold source of 10 °C. With the number of cycle increasing, the latent heats and phase change temperatures of the novel composite PCM were tested by DSC to determine the difference.

**Figure 4:**
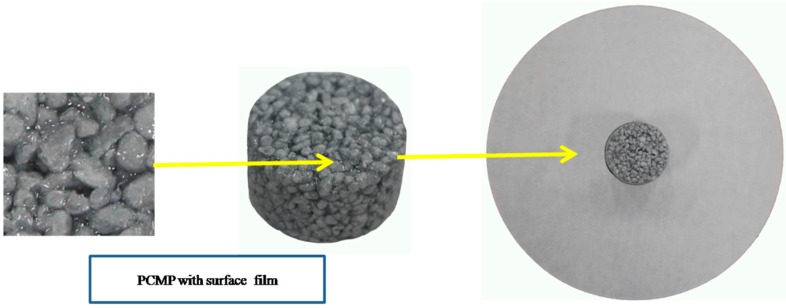
Schematic diagram of diffusion–effusion circle test.

The leakage test was conducted through diffusion–effusion circle method [37]. A circular sample of PCMP was laid on a circular region (ΦCR = 3 cm) in the centre of a filter paper, as shown in Figure 4. It can be found that the styrene-acrylic emulsion was completely encapsulated the PCM particle and can prevent the liquid PCM leaking from EP. Then, the filter paper with PCMP sample was heated for 2 h at the temperature 50 °C. The diameter of exudation region (ΦER) in the filter paper was tested to compare with ΦCR. The exudation percentage (*η*) can be calculated by Equation (3):

(3) $$\eta =\frac{\Phi ER-\Phi CR}{\Phi CR}\times 100\%$$

The mechanical test of the novel PCMP was conducted through the micro-control electronic universal testing machine (WDW-200 model, Changchun Kexin Test Instrument Co. Ltd., Changchun, China) with the accuracy of 0.01 kN and maximum testing force of 200 kN.

### 2.4. Thermal Performance Test

**Figure 5:**
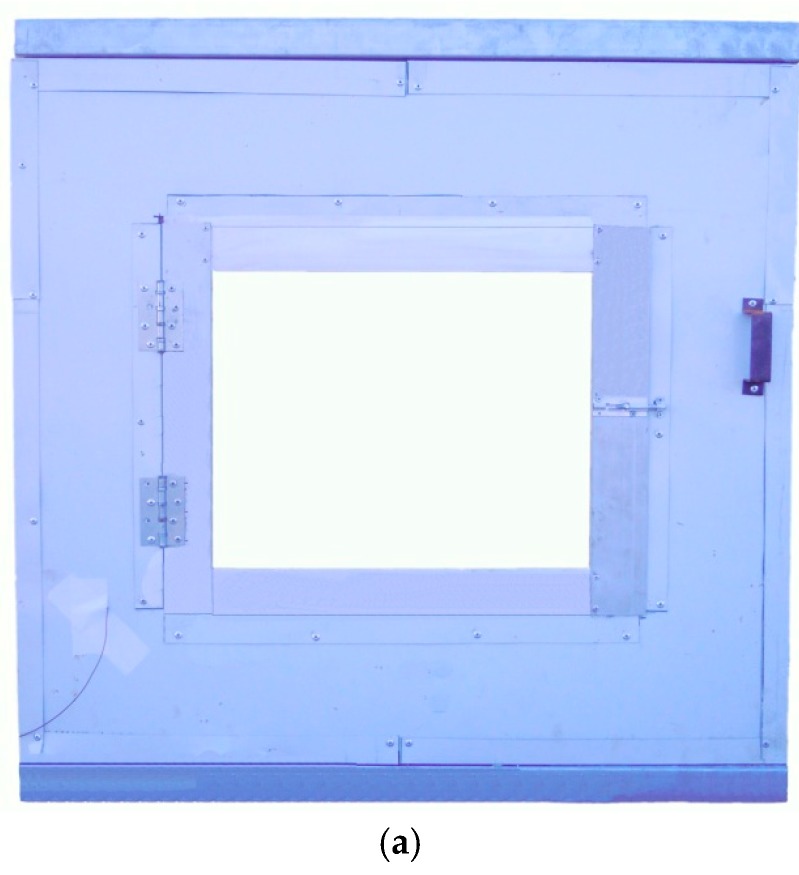

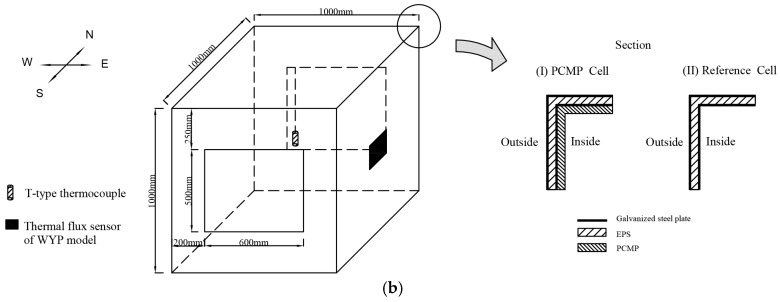
Introduction of tested cell and locations of measuring sensors. (**a**) External view of tested cell; (**b**) Schematic diagram of tested cell.

**Figure 6:**
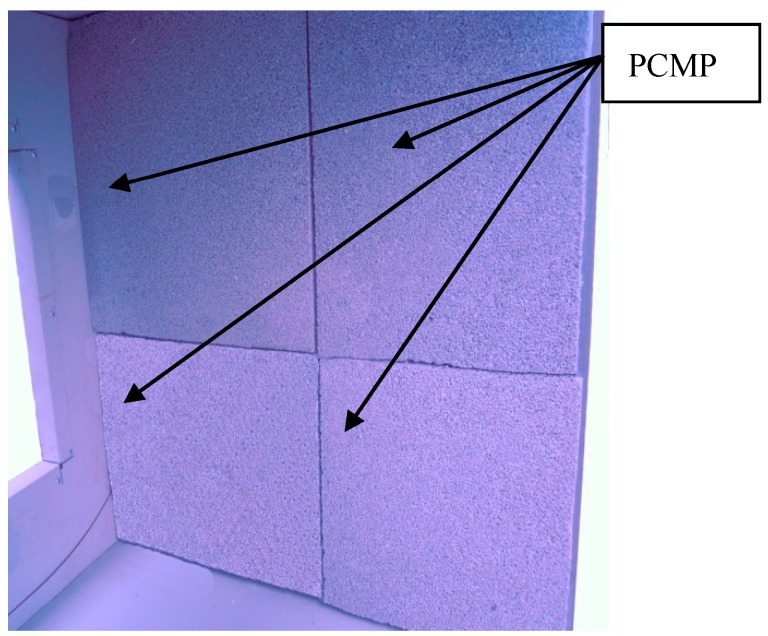
Drawing of PCMP installed into the inside wall of tested cell.

Two tested cells with size of 1 m × 1 m × 1 m have been fabricated by coloured steel sandwich insulation board (CSSIB) to test the thermal performance of the novel PCMP, as shown in Figure 5. The test cells were located in Tianjin, a middle latitude city in China, with a hot and dry summer but a cold winter [3]. The coloured steel sandwich insulation board is made of expandable polystyrene (EPS) foam board with two sides galvanized steel plate covered. Two windows with size of 60 cm × 50 cm were installed in the middle of south wall and north wall, and maintained closing with no sunshade in the experiment period. All the floor, ceiling and walls have the same thickness of 5 cm, and the floor was placed directly on the ground. One cell with PCMP (2 cm thickness, 3.45 m^2^ total area) installed on inside surfaces of the cell except for that of windows and floor was the PCM cell, and the other one without PCMP was as the reference cell. The important thermophysical parameters of the two cells are listed in Table 3. Figure 6 demonstrates the installation of PCMP on the PCM cell. The two cells were placed on the outdoor ground under the outdoor environmental conditions, in which the main influence factors were outdoor air temperature and solar radiation. T-type thermocouples with 1 mm diameter and accuracy ±0.4 K were instrumented in the middle of test cells to detect the temperature variation of inside cells. Thermal flux sensors of WYP model (standard errors ≤ 5%) were installed on the inside surface of the east wall of cells, so as to monitor the heat flux change. Solar radiometer (TBQ-2C model and ±2% accuracy) was used to record the solar radiation intensity. Besides, the same kind of thermocouple shadowed by trumpet-shaped tinfoil (avoiding the solar radiation affection) was chosen to measure the outdoor temperature [38]. The monitoring data from temperature and heat flux measurements were collected and transmitted to a computer by the data logger (Agilent BenchLink 34972A, Penang, Malaysia). Considering the outdoor thermal environment effect, the inside cell temperature and heat flux variation of the two cells were contrastively analysed in order to obtain the thermal effect of PCMP.

## 3. Results and Discussion

### 3.1. Thermal Property Analysis

**Figure 7:**
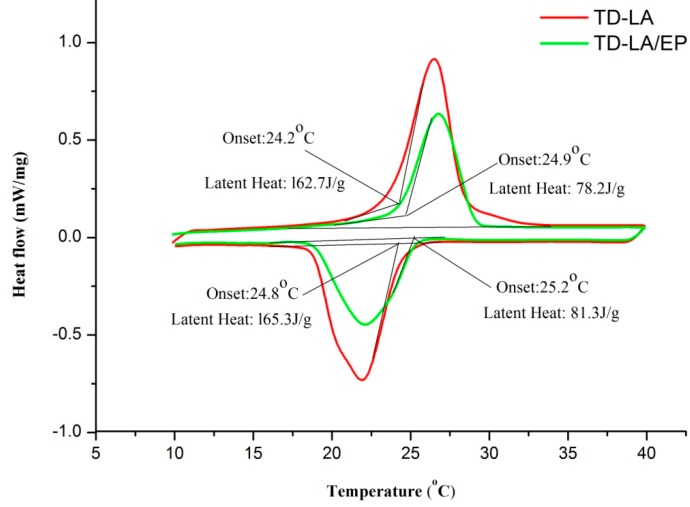
DSC curves of TD-LA and TD-LA/EP.

The DSC curves for TD-LA and TD-LA/EP are shown in Figure 7, in which the melting points of TD-LA and TD-LA/EP are 24.2 °C and 24.9 °C, respectively, and the detailed data are listed in Table 4. The change rate (CHR) was defined as Equation (4),

CHR = (T_i,TDLA_ − T_i,TDLA/EP_)/T_i,TDLA_ × 100% (4)

where T is the temperature, and subscript i represents the melting or freezing process. Both the pure TD-LA mixture and composite TD-LA/EP are fit for building energy storage. All the onset, peak andend points of TD-LA/EP are in close proximity to that of TD-LA, and the maximum CHR and difference of TD-LA/EP and TD-LA are 2.89% and 0.7 °C, respectively. The shift of phase change point is because of the weak attractive interaction of wall effect by the EP inner porous surfaces to PCM molecules, and this phenomenon has also been reported in reference [19].

The latent heats of TD-LA and TD-LA/EP are 162.7 J/g and 78.2 J/g in melting process and 165.3 J/g and 81.3 J/g in freezing process, respectively, which have been obtained through area integral in the DSC curves. It also can be found in Table 4 that the measured latent heat of TD-LA/EP was close to the value calculated by Equation (5).

(5) $$H_{CAL}=H_{p}\times M_{p}$$

where *H_CAL_*, *H_p_* and *M_P_* represent the calculating latent heat of TD-LA/EP, the latent heat of TD-LA/EP, and mass fraction of TD-LA in TD-LA/EP, respectively.

### 3.2. Construction Characterization and Mechanical Property Analysis

**Figure 8:**
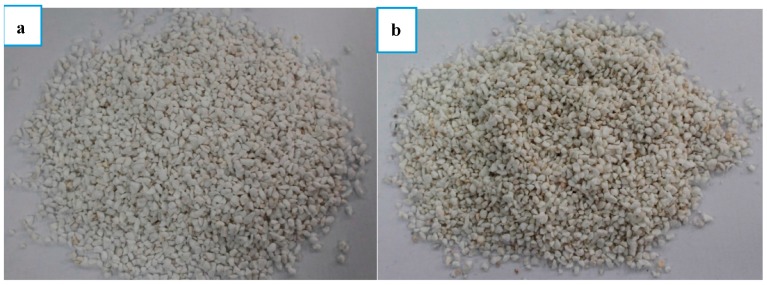
Images of: EP (**a**); and TD-LA/EP (**b**).

**Figure 9:**
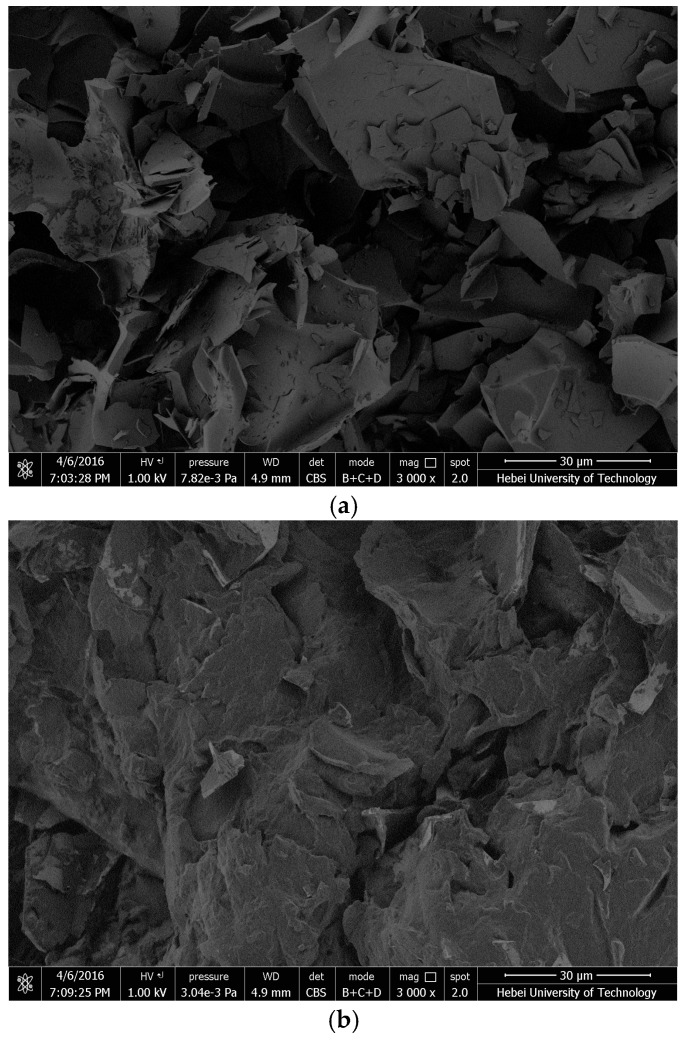
Morphological appearance of: EP (**a**); and TD-LA/EP (**b**).

The physical photos of expanded perlite before and after TD-LA vacuum impregnating are shown in Figure 8. Because of the impregnation only occurring in micropores, the overall appearances of EP and TD-LA/EP are almost the same, but differences can be found in Figure 9, in which it can be found that the expanded perlite has a compact internal structure and different shapes of lamina or lump. There are lots of irregular pores over the laminar and lumpy structures. SEM picture of EP illustrates that the expanded perlite has high porosity, great specific surface area and enough surface tension. On account of these favourable properties, expanded perlite is an appropriate supporting material that can absorb and maintain PCM. Figure 9b has showed that the microstructure of expanded perlite was changed after PCM absorption. Comparing with expanded perlite, the size and quantity of pores in TD-LA/EP were significantly decreased. It is due to that the pores in expanded perlite were successfully filled with TD-LA. Besides, the surface of composite PCM was rock-like and become much tighter than that of expanded perlite. Therefore, it is concluded that the PCM of TD-LA can be well absorbed into the micropores of expanded perlite.

The mechanical test result is listed in Table 5, in which the compressive strength values of the novel PCMP at three, seven and 15 days have been given. In the initial stage of the novel PCMP preparation, 2.09 N/m^2^ for compressive strength has indicated a low strength, but with the curing time increasing, the compressive strength values were enhance to be 4.21–4.23 N/m^2^. The final compressive strength value is close to the result of a kind of paraffin/EP-based PCM panel obtained in reference [39]. However, it is lower than the compressive strength of cement-based PCM panel, as seen in Table 5. Considering the novel PCMP is not functioned as the bearing wall, it still have qualified mechanical property, and a further study for enhancing the compressive strength of the novel PCMP will be conducted.

### 3.3. Thermal Conductivity Improvement Analysis

**Figure 10:**
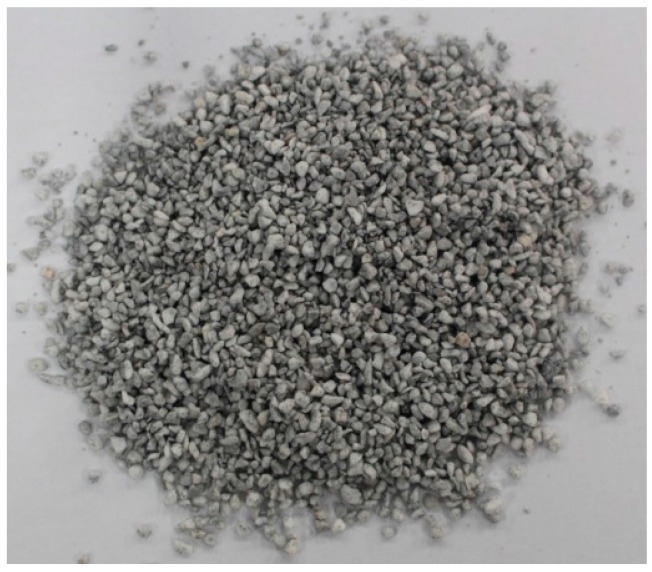
Appearance of TD-LA/EP-AP.

**Figure 11:**
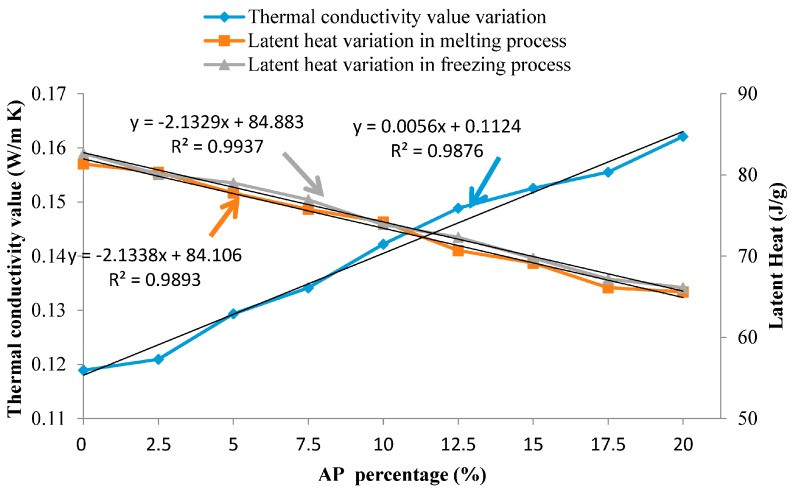
Variation comparison of thermal conductivity value and latent heat with different AP mass fraction for TD-LA/EP-AP.

The appearance of TD-LA/EP-AP is shown in Figure 10, and it can be found that TD-LA/EP-AP becomes dark in colour, because of the deep colour of the AP powder. With the AP mass fraction increasing, the thermal conductivity value is obviously enhanced, as shown in Figure 11, but the melting and freezing latent heats are gradually lowering, which is caused by PCM decreasing in total mass. Besides, a fact should be noted that the thermal conductivity value and latent heats are linearly changed with the AP percentage increasing. A similar result has been reported in [40,41]. Although the latent heat of the prepared composite PCM has been decreased, it is still in a high level, compared with that of other composite PCMs with similar phase change point in literatures, as shown in Table 6.

The heat transfer coefficients of PCMP samples with same size of 25 cm × 25 cm × 2 cm were obtained according to different AP mass fractions, and the results have been listed in Table 7. The heat transfer coefficients were also enhanced with the AP percentage increasing.

### 3.4. Thermal Reliability Analysis

An important property for PCM in actual application is the dependability of heat storage capacity. For PCM with higher cost than that of ordinary building materials, if PCM cannot maintain its original heat storage property during long time use, it will result in cost increasing but performance reducing. A thermal cycle test with many times should be conducted to study the thermal reliability of a new kind of composite PCM [19]. In this regard, the prepared novel composite PCM with 5 wt % AP (TD-LA/EP-5 wt % AP) was chosen to determine the dependability through measuring heat storage parameters after 1000 and 2000 thermal cycles.

**Figure 12:**
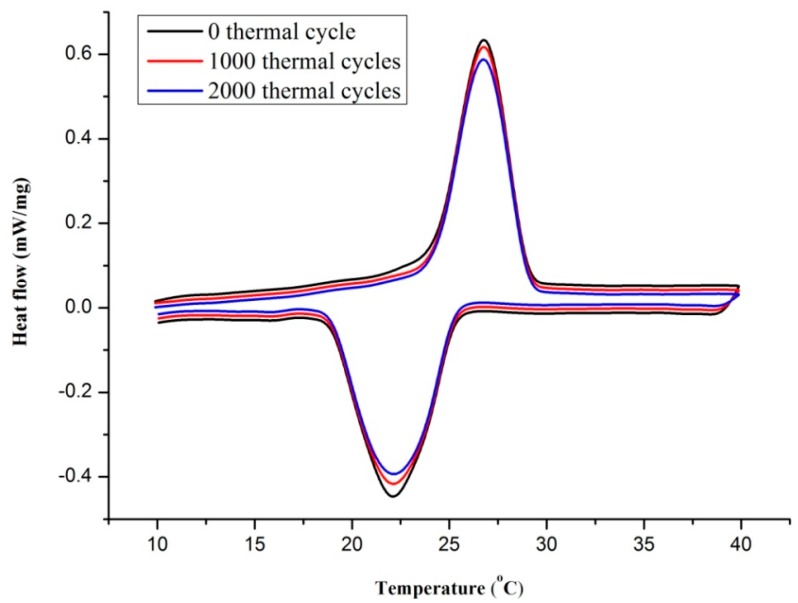
DSC curves of TD-LA/EP-AP after thermal cycling.

Figure 12 shows the DSC results of TD-LA/EP-5 wt % AP before and after thermal cycling. The coincident variation trends and no new heat peak and apparent amplitude variation have indicated that after many heat storing–releasing cycles, TD-LA/EP-AP still has good phase change properties. The detailed data are summarized in Table 8. After 1000 and 2000 thermal cycles, the melting and freezing points had changed little: 0.4 °C and −0.4 °C for 1000 cycles, and 0.2 °C and −0.3 °C for 2000 cycles. The latent heats of melting and freezing processes changed by −2.76% and −1.43% after 1000 cycles, and −4.36% and −3.92% after 2000 cycles, respectively. Based on the results in the literature [46,47], the small level changes for the phase change point and latent heat were acceptable for composite PCM using in buildings. Therefore, it can be obtained that the novel composite PCM has good thermal reliability and potential to be applied in building materials.

### 3.5. Leakage Analysis

**Figure 13:**
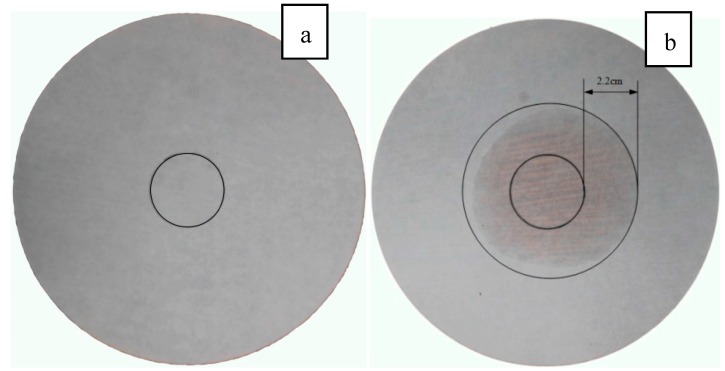
Leakage performance of TD-LA/EP-AP: (**a**) PCMP with surface film; and (**b**) PCMP without surface film.

The leakage of PCM in the building application can lead to several problems, such as destroying the mechanical properties of building materials, causing inconvenience to the residents, etc. Therefore, an application-oriented PCM in building should avoid the leakage issue. In this study, the diffusion–effusion circle method was used to test leakage performance of the novel PCMP (TD-LA/EP with 5 wt % AP), and the results are shown in Figure 13. The exudation percentage *η* of PCMP with surface film and without surface film were 0% and 73.3%, respectively. The fact that both the inside and outside circles have no leakage trace demonstrates that the surface film process is effective.

### 3.6. Thermal Performance Analysis

The thermal performance test has been conducted from 11 September 2015 to 13 September 2015 (late summer in test location), with no natural or mechanical night ventilation in experiment process. TD-LA/EP with 5 wt % AP was used as PCM for the novel PCMPs, which were bonded to the internal face of the test cell. Considering the combined influence of the outdoor air temperature and solar radiation, the outdoor sol-air temperature was used to show the outdoor temperature variation, which can be obtained by Equation (6) [48]:

*T_ost_* = *T_amb_* + *αI/h_out_* (6)

where *T_ost_* and *T_amb_* are the outdoor sol-air temperature (°C) and outdoor air temperature (°C), respectively; *α* is the solar absorption coefficient of the surface; I is the solar radiation (W/m^2^); and *h_out_* is the convective heat transfer coefficient (W/m^2^·K).

**Figure 14:**
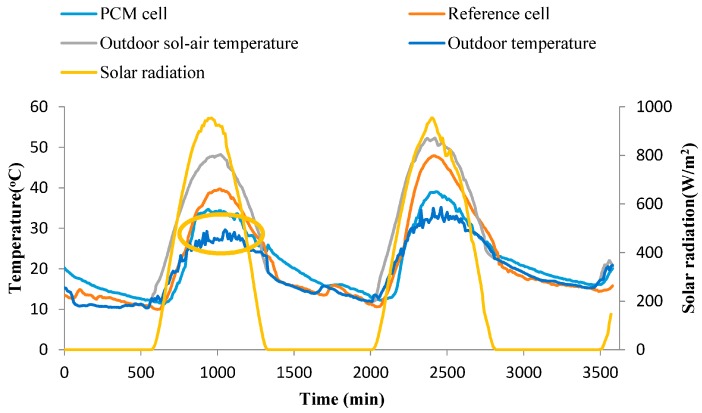
Temperature variations of PCM cell and reference cell during test period.

**Figure 15:**
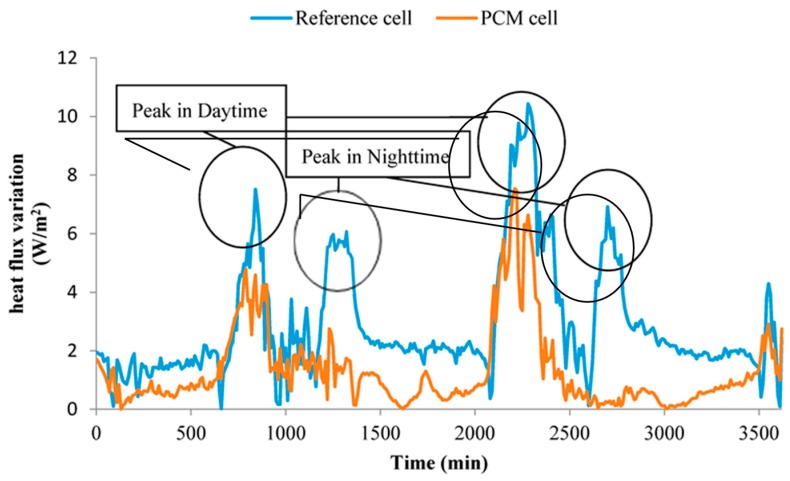
Heat flux variations of PCM cell and reference cell during test period.

Figure 14 presents the comparison of variation trends of PCM and reference cells under the real outdoor environmental conditions during 2.5 days (3600 min). It is obvious that the PCM cell has better thermal performance, especially in daytime. The results are summarized in Table 9. In detail, three points can be highlighted in terms of experiment results:

(1) In day time, when the indoor temperature is higher than the melting temperature of TD-LA/EP-5 wt % AP, PCMP started to absorb the heat from inside cell to restrain the indoor temperature rise. The peak temperature and mean inside temperatures of PCM cell were 6.96 °C and 4.10 °C lower than that of reference room in daytime (6:00–18:00), respectively. On the first day, a relative smooth temperature section emerged at noon. Temperatures in this smooth section were much higher than the melting point of PCMP, indicating the latent heat storage of PCMP was possibly finished, and the sensible heat storage of PCMP played a leading role in this section. Similarly, the peak temperature decreasing may also mainly result from the sensible heat storage of PCMP. Therefore, the heat storage of PCMP included sensible heat storage and latent heat storage, and they simultaneously effected in the inside temperature decreasing. The ratio of latent/sensible heat storage will be studied in detail in the future.
(2) For night time (18:00–6:00), because of ambient temperature decreasing and without solar radiation, the indoor temperature was reduced, and PCMP began to release the heat stored during daytime if the indoor temperature was lower than the freezing point of PCM. Therefore, mean inside temperature of PCM cell was 2.43 °C higher than that of reference cell. In other words, the heat absorbing and releasing by prepared PCMP can effectively reduce the indoor temperature fluctuation.
(3) The heat flux variations of PCM cell wall and reference cell wall are shown in Figure 15, in which the heat flux value indicated the heat exchange capacity between cells and external environment. As listed in Table 9, the peak and mean heat fluxes of PCM cell were 2.81 W/m^2^ and 1.64 W/m^2^ smaller than that of reference cell, respectively. Besides, the fluctuation of heat flux values of PCM cell were smaller than that of reference cell during all the experiment period. The small heat flux value and range have showed that the prepared PCMP can reduce the influence of external disturbance on the inside thermal environment.

The greenhouse effect through windows and the low thermal mass of the cell envelope resulted in the inside temperature of cells being higher than the outdoor temperature at noon. However, considering the results obtained by a comparison method (PCMP cell vs. reference cell) under the same outdoor conditions, the effect of this experiment limitation can be lowered to a minimum level. The other experiment limitation is the reference cell did not have a substitute panel with the same thickness of PCMP, which leads to a difference in the thermal transmission of the two cells. Thus, the current study qualitatively obtained the thermal performance of PCMP through the contrasting approach with and without PCMP installed in the cells. The next study will focus on the quantitative study of the comparison of PCMP and other building materials in the same condition of thermal transmission.

## 4. Conclusions

In this study, a composite PCM, consisting of PCM of TD-LA binaryeutectic mixture, supporting material of EP, thermal conductive promoter of AP and surface film of styrene-acrylic emulsion, were fabricated using vacuum impregnation and mixture stirring methods. Then, a novel PCMP was prepared through compression moulding to be used for building energy storing/releasing. The following conclusions can be deduced based on all results:

(1) TD-LA/EP has melting/freezing temperature and latent heat of 24.9 °C/25.2 °C and 78.2 J/g/81.3 J/g, respectively, which are suitable for building heat storage.
(2) With the mass ratio of thermal conductivity promoter of AP increasing, the thermal conductivity value has been enhanced linearly, but the latent heat was linearly decreased. It seems that an AP mass ratio range of 5–15 wt % was reasonable to obtain a high thermal conductivity and while maintain a large latent heat.
(3) The leakage test using diffusion–effusion circle method indicated that the film attached on the surface of composite PCM particles was an effective solution for the leaking issue of composite PCMs.
(4) The results of mechanical test and thermal cycling test demonstrated that the fabricated PCMP have good mechanical property and thermal reliability for building application.
(5) The thermal performance test has been conducted through two cells, PCM cell and reference cell, with lightweight envelope (CSSIB) under the same outdoor climatic condition during 2.5 days. Compared with the result of reference cell in thermal performance test, the cell with PCMP was on average 4.10 °C cooler during day time and 2.43 °C warmer during night time, and had lower heat flux value. It also was found that both the latent and sensible heat storage capacities of PCMP functioned in indoor temperature decreasing. Because was PCMP installed in PCM cell, the two cells had different thermal transmittance and thermal mass. Thus, a qualitative thermal performance of PCMP was obtained at present stage, and an in-depth contrastive study for PCMP performance under the same thermal transmittance and thermal mass in building envelopes will be carried out in the next stage.
(6) Based on all results, it can also be concluded that the developed novel PCMP can be used as building heat storage panel in the wall inside to diminish the building energy consumption and adjust the indoor comfort.

## Figures and Tables

**Table 1:** Chemical composition of EP.

Constituent	SiO_2_	Al_2_O_3_	K_2_O	Na_2_O	MgO	CaO
Mass percent (wt %)	73	18	4.3	4.1	0.4	0.2

**Table 2:** Comparison of measured and calculated melting temperatures and mass percentages for TD and LA.

PCM	Melting Point (°C)		Mass Percentage in Binary Eutectic Mixture (%)
	Measured Value	Predicted Value	
TD	37.00	–	53.60
LA	43.20	–	46.40
TD-LA	24.2	25.8	100

**Table 3:** Properties of tested cell envelope.

Item	Reference Cell				PCMP Cell			
	Wall	Ceiling	Floor	Window	Wall	Ceiling	Floor	Window
Structure	CSSIB	CSSIB	CSSIB	Single glazing Aluminium alloy	CSSIB + PCMP	CSSIB + PCMP	CSSIB	Single glazing Aluminium alloy
Heat transfer coefficient (W/m^2^·K)	0.763	0.763	0.763	6.4	0.645	0.645	0.763	6.4
Thickness (mm)	50	50	50	6	0.645	0.645	50	6
Reflectivity (%)	–	–	–	89	–	–	–	89
Transmittance (%)	–	–	–	8	–	–	–	8

**Table 4:** Thermal properties for the TD-LA and TD-LA/EP.

PCM	Phase Change Point (°C)						Latent Heat (J/g)		
	Melting			Freezing			Melting	Freezing	Calculating
	Onset	Peak	End	Onset	Peak	End			
TD-LA	24.2	26.5	28.8	24.8	22	19.9	162.7	165.3	–
TD-LA/EP	24.9	26.9	29	25.2	22.3	20.1	78.2	81.3	81.4
CHR (%)	2.89%	1.51%	0.69%	1.61%	1.36%	1.01%	–	–	–

**Table 5:** Compressive strength results of the novel PCMP and several composites PCMs in references.

Item	Curing Time (Days)	Compressive Strength (N/m^2^)	Reference
Paraffin/expanded perlite/cement mortar	7	4.04	[29]
	28	7.53	
PCM powder/cement mortar	7	3.62	[27]
	28	7.16	
Paraffin/expanded perlite	–	4.61	[39]
TD-LA/EP-AP	3	2.09	This study
	7	4.21	
	15	4.23	

**Table 6:** Comparison of heat storage parameters of the novel composite PCM with that of some PCMs in another researches.

Item	Melting Point (°C)	Freezing Point (°C)	Latent Heat of Melting (J/g)	Latent Heat of Freezing (J/g)	Reference
Bentonite/dodecanol/5 wt % EG	22.16	21.05	57.84	55.45	[42]
Bentonite/heptadecane/5 wt % EG	22.09	21.53	34.05	32.43	[42]
Dodecanol/cement	21.6	–	18.39	–	[43]
Capric–stearic acid/gypsum	23.8	23.9	49	–	[44]
Stearic–palmitic–myristic–lauric acid/sludge ceramsite	26.6	–	47.1	–	[45]
Pumice/capric–palmitic acid	23.13	21.65	56.45	55.4	[46]
Pumice/heptadecane	22.18	21.14	72.38	70.24	[46]
Pumice/dodecanol	23.27	20.98	67.32	66.48	[46]
TD-LA/EP-5 wt % AP	24.5	25.4	77.78	79.02	This study
TD-LA/EP-10 wt % AP	24.7	25.6	74.22	73.89	This study
TD-LA/EP-15 wt % AP	24.3	24.9	69.15	69.75	This study

**Table 7:** Heat transfer coefficients with different AP percentage.

PCMP Samples	Sample 1#	Sample 2#	Sample 3#	Sample 4#	Sample 5#	Sample 6#	Sample 7#	Sample 8#	Sample 9#
AP percentage (%)	0	2.5	5	7.5	10	12.5	15	17.5	20
Heat transfer coefficient (W/m^2^·K)	2.38	2.42	2.59	2.68	2.84	2.98	3.05	3.11	3.24

**Table 8:** Comparison of heat storage parameters of TD-LA/EP-AP before and after 1000 and 2000 cycles.

PCM	Number of the Cycle	Melting Onset Point (°C)	Freezing Onset Point (°C)	Melting Latent Heat (J/g)	Freezing Latent Heat (J/g)
TD-LA/EP-5 wt % AP	0 cycle	24.5	25.4	77.78	79.02
	1000 cycles	24.9	25	75.63	77.89
	2000 cycles	25.1	24.7	74.39	75.92

**Table 9:** Comparison of detailed thermal data for PCM and reference cells during 48 h.

Item	PCM Cell	Reference Cell
Peak temperature (°C)	36.85	43.81
Mean temperature of day time (°C)	28.11	32.21
Mean temperature of night time (°C)	16.3	13.87
Peak heat flux (W/m^2^)	6.16	8.97
Mean heat flux (W/m^2^)	1.44	3.08
